# “Spaghetti twisting” technique: a novel method of catching pacemaker leads using a needle's eye snare

**DOI:** 10.1002/ccr3.1050

**Published:** 2017-06-23

**Authors:** Tsuyoshi Isawa, Takashi Yamada, Taku Honda, Kazuhiro Yamaya, Tatsushi Ootomo

**Affiliations:** ^1^ Department of Cardiology Sendai Kousei Hospital Sendai Japan; ^2^ Department of Cardiology Takaishi Fujii Cardiovascular Hospital Takaishi Japan; ^3^ Department of Cardiovascular Surgery Sendai Kousei Hospital Sendai Japan

**Keywords:** femoral approach, needle's eye snare, “Spaghetti twisting” technique

## Abstract

The needle's eye snare has become an indispensable tool in contemporary pacemaker lead extraction techniques. Here, we present a modified method of using the needle's eye snare, named “spaghetti twisting” technique, to catch and secure pacemaker leads, which would help operators catch and secure leads much easily.

## Introduction

Superior lead extraction has often been used as a primary approach for pacemaker lead removal 1. However, approaching lead removal via the femoral vein is necessary in cases where leads have been purposefully cut or where lead fragments have been abandoned during a previous removal attempt. A number of tools and techniques have been developed for extracting leads via the femoral vein. The needle's eye snare device (Cook Medical Inc., Bloomington, IN, USA) is one of these tools 2. However, it is sometimes difficult to catch a lead without a free end in the right atrium (RA). Here, we describe a modified method of using the needle's eye snare to catch and secure pacemaker leads.

## Case presentation

**Figure 1:**
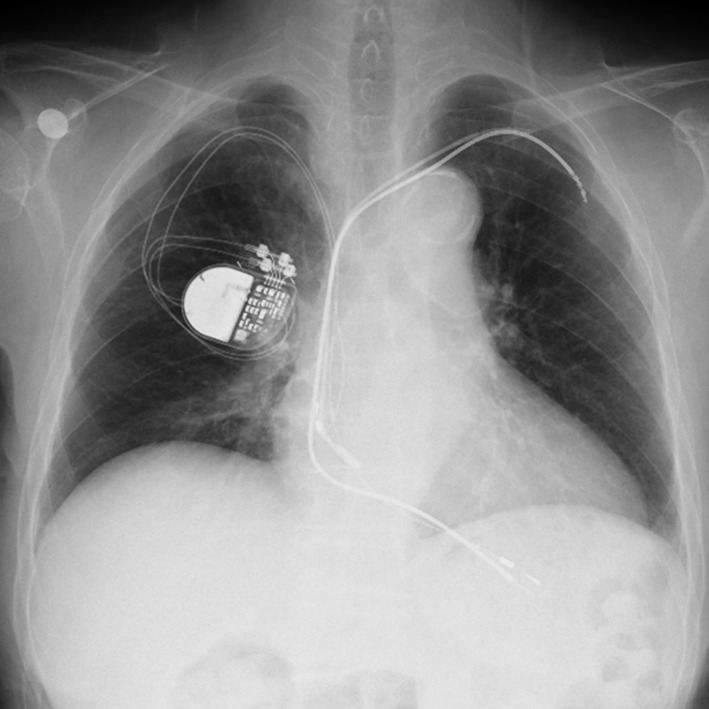
Chest X‐ray on admission showing the older two leads at the left pectoral region, confirming the cutting of proximal segments during a previous procedure, and the newer two leads on her right side.

**Figure 2:**
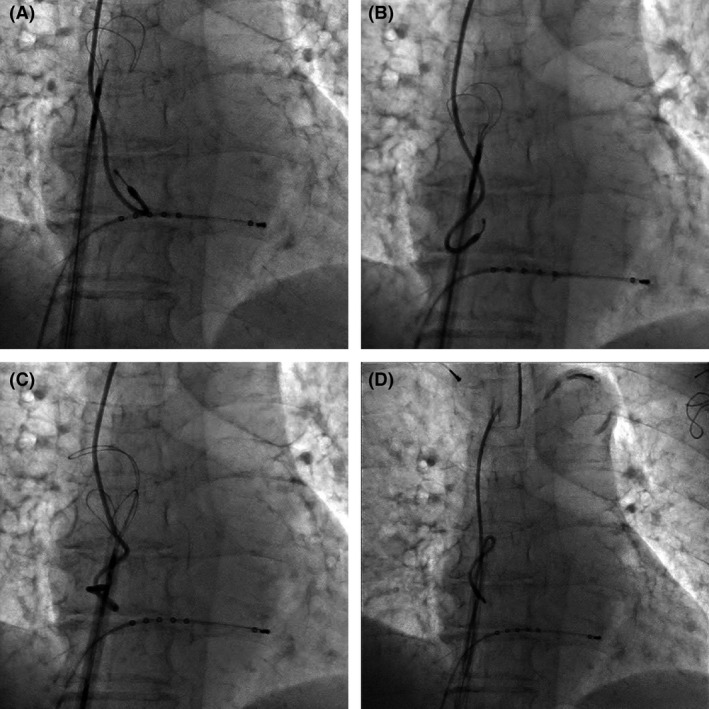
(A) The snare was gently advanced into the superior vena cava. (B) The snare twisted the lead around it like “twisting spaghetti around a fork.” (C) A threader is extended in between the struts of the snare on the opposite site of the body. (D) The lead was extracted into the sheath.

**Figure 3:**
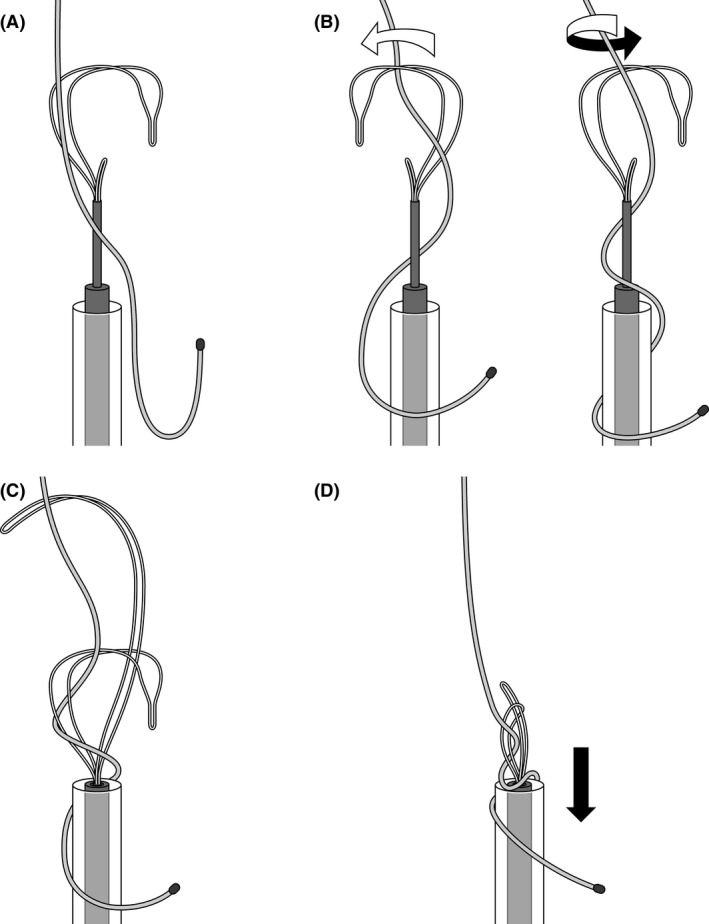
The schema of “spaghetti twisting” technique.

**Figure 4:**
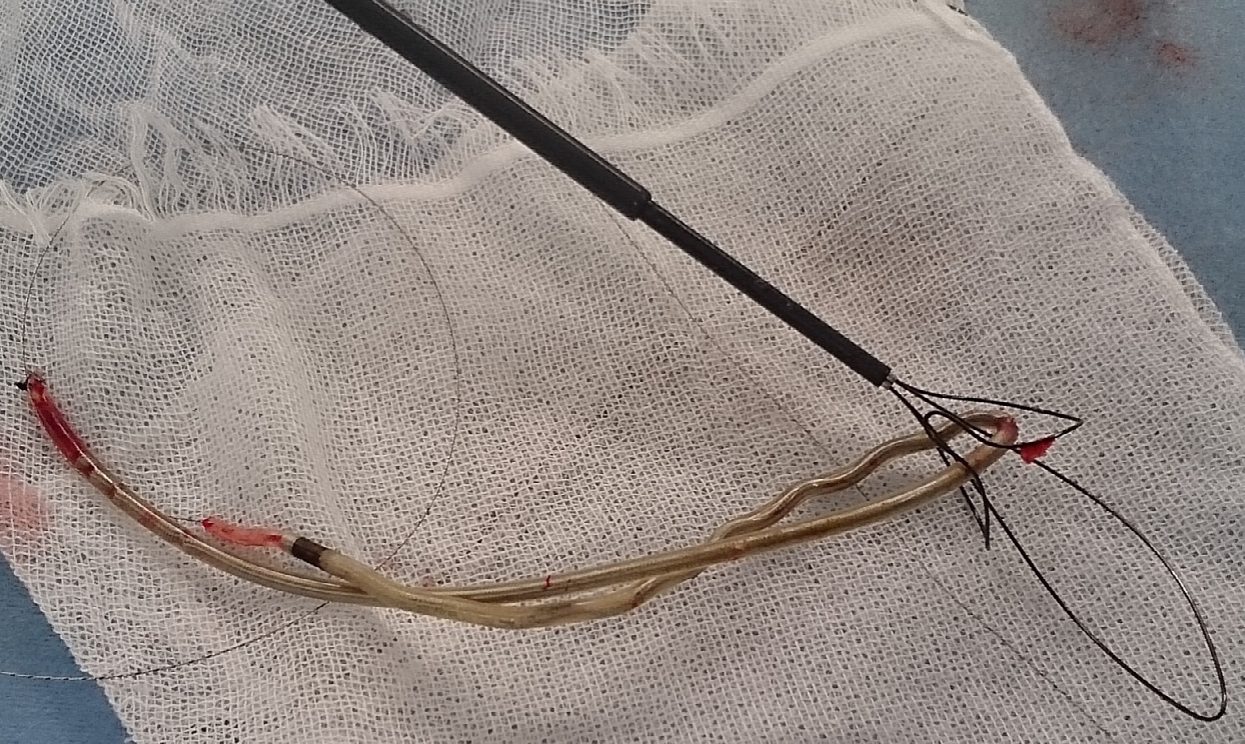
Extracted lead. The needle's eye snare retriever pushed out of the sheath.

An 89‐year‐old woman with sick sinus syndrome was referred to our institute for an ulcer located at her pacemaker pocket site in the left pectoral region, which had been present for 2 weeks. She had been fitted with a dual‐chamber pacemaker with two passive leads (Bipolar Membrane EX model 1474 T, Pacesetter, Jarfalla, Sweden, for the ventricular lead and Bipolar Membrane II model 1452 T, Pacesetter, for the atrial lead) 12 years ago in her left pectoral region. One year prior to this admission, she had developed a pocket abscess and had undergone an unsuccessful lead extraction procedure at another hospital, during which attempts to remove the leads by simple traction failed. Subsequently both of the proximal lead segments were pulled out and cut, leaving a short length of looped leads in the pacemaker pocket. A new pacemaker was then implanted on the opposite site. Her presenting symptoms were ulceration at the pacemaker pocket site with serous drainage. Physical examination revealed the following vital signs: body temperature, 36.9°C; pulse, 70 beats/min, regular; and blood pressure, 131/82 mm Hg. An electrocardiogram revealed a pacing rhythm of 70 beats/min. Although blood cultures remained negative, wound cultures were positive for *Escherichia coli*. A chest X‐ray revealed the location of the older two leads to be at her left pectoral region, confirming the proximal segments had been cut in a previous procedure; the newer two leads on her right side could also be seen (Fig.**** 1). After formal heart team discussion, the newer and older leads were scheduled to be fully extracted by simple traction, a laser sheath (SLS II, Spectranetics, Colorado Springs, CO, USA), and a needle's eye snare (Cook Medical Inc., Bloomington, IN, USA). The lead extraction procedure was performed in a hybrid operating room under general anesthesia by both interventional cardiologists and cardiac surgeons, with extracorporeal circulation on standby. Initially, the newer leads implanted from the right subclavian vein were successfully extracted using simple traction with the help of a locking stylet (Liberator, Cook Medical Inc.). Then, the infected pocket in the left pectoral region was widened, and the pocket was heavily debrided of all inflammatory tissue. The free end of the ventricular lead in the left pectoral region was able to be secured and removed using a locking stylet (Lead Locking Device, Spectranetics) and a laser sheath. However, the atrial lead's cut point was too deep in the pacemaker pocket region, and it was impossible to grasp its free end via the subclavian route. Therefore, right femoral vein access was obtained through which a 16‐Fr introducer sheath was advanced over a 0.035‐inch guidewire into the RA. The needle's eye snare retriever (13 mm) and the accompanying 12‐Fr sheath were introduced through the 16‐Fr sheath to snare the lead in the RA. However, several attempts failed to grasp the lead body using the needle's eye snare. Therefore, the snare was gently advanced into the superior vena cava (SVC) and the lead twisted around like “twisting spaghetti around a fork.” After the snare had wound up the lead, a second loop or threader secured the snare around the lead body. Once secured, the 12‐Fr sheath was pulled into the 16‐Fr introducer sheath. The 16‐Fr introducer sheath was advanced over the doubled‐up leads, which were successfully extracted into the sheath (Figs. 2, 3, 4, Movies S1, S2 and S3). The procedure was completed without any complications. The patient complained of no symptoms related to bradycardia after a pacemaker was removed. The rest of the patient's stay in hospital was uneventful, and she was discharged 2 weeks after the procedure.

## Discussion

In this report, we presented a modified method of using the needle's eye snare, where twirling the needle's eye snare in the SVC facilitates the catching and securing of a pacemaker lead.

The needle's eye snare is a tool commonly used for lead extraction using the femoral vein 2. It gives the advantage of being able to grasp a lead that does not have a free end. The most common way to grasp a lead using the needle's eye snare is as follows: the snare is positioned over the leads, and a threader is extended in between the struts of the snare on the opposite site of the body. When the 16‐Fr sheath is advanced, the lead becomes trapped between both loops 3. While the use of needle's eye snare as mentioned above is usually very successful, it does not always work. Instead, the “spaghetti twisting” technique is, in our opinion, more successful and therefore should be used widely.

There are several advantages in utilizing this technique. Firstly, it is an operator‐friendly technique. The femoral approach for lead extraction is typically used as a bailout procedure. Therefore, there are only a few cases where lead extraction via the femoral vein has been performed, and it is difficult to gain much experience with a femoral approach using a needle's eye snare. Further to this point, this “spaghetti twisting” technique is easy to master and enables the operators to twist and catch a lead easily despite the lack of operator experience. Secondly, this technique makes catching a lead as quick as possible even in patients with large right atria. The smaller sized needle's eye snare retriever (13 mm) and the larger one (20 mm) are now available. Without the application of the “spaghetti twisting” method, it is necessary to change the smaller needle's eye snare for the larger one in cases of patients with large right atria, as using the smaller snare within the RA makes capture more difficult. However, if the “spaghetti twisting” technique is used, the larger RA has little influence on the time required for catching a lead because the snare is advanced into the SVC, which is narrower than the RA. And finally, this technique combined with straight sheaths enables retrieval of leads in more of a coaxial position and provides a stronger pulling power, resulting in more successful removal.

There are some technical considerations to accommodate when employing the “spaghetti twisting” technique. First, a previous report revealed that the complication rate with the needle's eye snare approach is low (0.7%) 3. However, this “spaghetti twisting” technique requires manipulation into the SVC, which can cause vascular tearing, requiring immediate surgery. Therefore, not only is very gentle manipulation of the snare essential, but also active surgical backup with standby extracorporeal circulation in case of vascular tear should be considered mandatory for the application of this technique during percutaneous lead extraction. Second, this technique cannot be applied when leads are bounded to SVC or other leads. Therefore, intracardiac echocardiography should be performed before introducing the needle's eye snare to identify whether the leads are adherent to SVC or other leads. Third, while applying this technique to lead extraction, the needle's eye snare must be twisted several times around the lead to catch the lead, and it is not always possible to secure the lead at that time with the inner loop because the outer loop of the needle's eye snare gets distorted or multiple loops of the lead are captured at once, and the 16‐Fr outer sheath sometimes cannot be advanced over the lead. Therefore, an 18 Fr size outer sheath at minimum, in our opinion, is necessary despite the increase in the access site bleeding risk. Finally, we cannot help using only simple traction while using this technique because multiple loops of the lead are usually captured at once, making it impossible to secure the lead in a classic mode, namely to close the snare and ensure a firm grasp of the lead by advancing the inner sheath. Consequently, the disruption of fibrous binding tissues using both the inner and outer sheath would become impossible.

## Conclusions

The modified method of the needle's eye snare, as mentioned above, facilitates catching and securing pacemaker leads via femoral access and can be broadly applied to lead extraction.

## Conflict of interest

Dr. Yamada has reported speaker fees from DVx Inc. Tokyo, Japan. All other authors have reported no financial relationships or conflict of interests regarding the content of the manuscript.

## Authorship

TI, TH, KY, and TO: collected and analyzed the data. TI: wrote the article. TY: had the idea for the article and revised it critically.

## Supporting information


**Movies S1.** The snare was gently advanced into the superior vena cava. The snare twisted the lead around it like “twisting spaghetti around a fork.” A threader is extended in between the struts of the snare on the opposite site of the body.

**Movies S2.** The lead was extracted into the sheath.

**Movies S3.** The lead was extracted into the sheath.
